# Graph-distance distribution of the Boltzmann ensemble of RNA secondary structures

**DOI:** 10.1186/1748-7188-9-19

**Published:** 2014-09-11

**Authors:** Jing Qin, Markus Fricke, Manja Marz, Peter F Stadler, Rolf Backofen

**Affiliations:** 1 Department of Mathematics and Computer Science, Campusvej 55, DK-5230, Odense M, Denmark; 2 Max Planck Institute for Mathematics in the Sciences, Inselstraße 22, D-04103 Leipzig, Germany; 3 Bioinformatics/High Throughput Analysis Faculty of Mathematics und Computer Science Friedrich-Schiller-University, Leutragraben 1, D-07743 Jena, Germany; 4 Department of Computer Science, Chair for Bioinformatics, University of Freiburg, Georges-Koehler-Allee 106, D-79110 Freiburg, Germany; 5 Center for Biological Signaling Studies (BIOSS), Albert-Ludwigs-Universität, Freiburg, Germany; 6 Bioinformatics Group, Department of Computer Science, and Interdisciplinary Center for Bioinformatics, University of Leipzig, Härtelstrasse 16-18, D-04107 Leipzig, Germany; 7 Fraunhofer Institut for Cell Therapy and Immunology, Perlickstraße 1, D-04103 Leipzig, Germany; 8 Institute for Theoretical Chemistry, University of Vienna, Währingerstrasse 17, A-1090 Vienna, Austria; 9 Santa Fe Institute, 1399 Hyde Park Rd., NM87501 Santa Fe, USA

**Keywords:** Graph-distance, Boltzmann distribution, Partition function, Pre-mRNA splicing, smFRET

## Abstract

**Background:**

Large RNA molecules are often composed of multiple functional domains whose spatial arrangement strongly influences their function. Pre-mRNA splicing, for instance, relies on the spatial proximity of the splice junctions that can be separated by very long introns. Similar effects appear in the processing of RNA virus genomes. Albeit a crude measure, the distribution of spatial distances in thermodynamic equilibrium harbors useful information on the shape of the molecule that in turn can give insights into the interplay of its functional domains.

**Result:**

Spatial distance can be approximated by the graph-distance in RNA secondary structure. We show here that the equilibrium distribution of graph-distances between a fixed pair of nucleotides can be computed in polynomial time by means of dynamic programming. While a naïve implementation would yield recursions with a very high time complexity of *O*(*n*^6^*D*^5^) for sequence length *n* and *D* distinct distance values, it is possible to reduce this to *O*(*n*^4^) for practical applications in which predominantly small distances are of of interest. Further reductions, however, seem to be difficult. Therefore, we introduced sampling approaches that are much easier to implement. They are also theoretically favorable for several real-life applications, in particular since these primarily concern long-range interactions in very large RNA molecules.

**Conclusions:**

The graph-distance distribution can be computed using a dynamic programming approach. Although a crude approximation of reality, our initial results indicate that the graph-distance can be related to the smFRET data. The additional file and the software of our paper are available from http://www.rna.uni-jena.de/RNAgraphdist.html.

## Background

The distance distribution within an RNA molecule is of interest in various contexts. Most directly, the question arises whether panhandle-like structures (in which 3’ and 5’ ends of long RNA molecules are placed in close proximity) are the rule or an exception. Panhandles have been reported in particular for many RNA virus genomes. Several studies [1-4] agree based on different models that the two ends of single-stranded RNA molecules are typically not far apart. On a more technical level, the problem to compute the partition function over RNA secondary structures with given end-to-end distance *d*, usually measured as the number of external bases (plus possibly the number of structural domains) arises for instance when predicting nucleic acid secondary structure in the presence of single-stranded binding proteins [5] or in models of RNA subjected to pulling forces (e.g. in atom force microscopy or export through a small pore) [6-8]. It also plays a role for the effect of loop energy parameters [9].

In contrast to the end-to-end distance, the graph-distance between two *arbitrarily* prescribed nucleotides in a larger RNA structure does not seem to have been studied in any detail. However, this is of particular interest in the analysis of single-molecule fluorescence resonance energy transfer (smFRET) experiments [10]. This technique allows to monitor the distance between two dye-labeled nucleotides and can reveal details of the kinetics of RNA folding in real time. It measures the non-radiative energy transfer between the dye-labeled donor and acceptor positions. The efficiency of this energy transfer, *E*_*fret*_, strongly depends on the spatial distance *R* according to $E_{\text{fret}}=R_{0}^{6}/(R_{0}^{6}+R^{6})$. The Förster radius *R*_0_ sets the length scale, e.g. *R*_0_≈54 Å for the Cy3-Cy5 dye pair. A major obstacle is that, at present, there is no general and efficient way to link smFRET measurements to interpretations in terms of explicit molecular structures. To solve this problem, a natural first step is to compute the distribution of spatial distances for an equilibrium ensemble of 3D structures. Since this is not feasible in practice despite major progress in the field of RNA 3D structure prediction [11], we can only resort to considering the graph-distances on the ensemble of RNA secondary structures instead. From a computer science point of view, furthermore, we show here that the distance distribution can be computed exactly using a dynamic programming approach. Although a crude approximation of reality, our initial results indicate that the graph-distance can be related to the smFRET data such as those reported by [12] and help to explain effects of RNA structures in pre-mRNA splicing and viral subgenomic RNA species.

## Theory

### RNA secondary structures

An RNA secondary structure is a vertex labeled outerplanar graph *G*(*V*,*x*,*E*), where *V *= {1,2,…,*n*} is a finite *ordered* set (of nucleotide positions) and *x *: {1,2,…,*n*} → {A,U,G,C},*i *↦ *x*_*i*_ assigns to each vertex at position *i* (along the RNA sequence from 5’ to 3’) the corresponding nucleotide *x*_*i*_. We write *x *= *x*_1_…*x*_*n*_ for the *sequence* underlying secondary structure and use *x*[ *i*…*j*] = *x*_*i*_…*x*_*j*_ to denote the *subsequence* from *i* to *j*. The edge set *E* is subdivided into backbone edges of the form {*i*,*i *+ 1} for 1 ≤ *i*<*n* and a set *B* of base pairs satisfying the following conditions: 

(i) If {*i*,*j*} ∈ *B* then *x*_*i*_*x*_*k *_∈ {GC,CG,AU,UA,GU,UG};

(ii) If {*i*,*j*} ∈ *B* then |*j *- *i*| > 3;

(iii) If {*i*,*j*},{*i*,*k*} ∈ *B* then *j* = *k*;

(iv) If {*i*,*j*},{*k*,*l*} ∈ *B* and *i *< *k *< *j* then *i *< *l *< *j*.

The first condition allows base pairs only for Watson-Crick and GU base pairs. The second condition implements the minimal steric requirement for an RNA to bend back on itself. The third condition enforces that *B* forms a matching in the secondary structure. The last condition (nesting condition) forbids crossing base pairs, i.e. pseudoknots.

The nesting condition results in a natural partial order in the set of base pairs *B* defined as {*i*,*j*} ≺ {*k*,*l*} if *k *< *i *< *j *< *l*. In particular, given an arbitrary vertex *k*, the set *B*_*k *_= {{*i*,*j*} ∈ *B*|*i *≤ *k *≤ *j*} of base pairs enclosing *k* is totally ordered. Note that *k* is explicitly allowed to be incident to its enclosing base pairs. A vertex *k* is *external* if *B*_*k *_= *∅ *. A base pair {*k*,*l*} is *external* if *B*_*k *_= *B*_*l *_= {{*k*,*l*}}.

Consider a fixed secondary structure *G*, for a given base pair {*i*,*j*} ∈ *B*, we say a vertex *k* is *accessible* from {*i*,*j*} if *i *< *k *< *j* and there is no other pair {*i*^′^,*j*^′^} ∈ *B* such that *i *< *i*^′ ^< *k *< *j*^′ ^< *j*. The unique subgraph ${ℒ}_{i,j}$ induced by *i*, *j*, and all the vertices accessible from {*i*,*j*} is known as the *loop* of {*i*,*j*}. The *type* of a loop ${ℒ}_{i,j}$ is unique determined depending on whether {*i*,*j*} is external or not, and the numbers of unpaired vertices and base pairs. For details, see [13]. Each secondary structure *G* has a unique set of loops $\left\{{ℒ}_{i,j}|\{i,j\}\in B\right\}$, which is called the *loop decomposition* of *G*. The free energy *f*(*G*) of a given secondary structure, according to the standard energy model [14], is defined as the sum of the energies of all loops in its unique loop decomposition.

The relative location of two vertices *v* and *w* in *G* is determined by the base pairs *B*_*v*_ and *B*_*w*_ that enclose them. If *B*_*v *_∩ *B*_*w *_≠ *∅*, there is a unique ≺-minimal base pair {*i*_*v*,*w*_,*j*_*v*,*w*_} that encloses both vertices and thus a uniquely defined loop ${ℒ}_{\left\{i_{v,w},j_{v,w}\right\}}$ in the loop associated with *v* and *w*. If *B*_*v *_∖ *B*_*w *_= *∅ * or *B*_*w *_∖ *B*_*v *_= *∅* then *v* or *w* is unpaired and part of ${ℒ}_{\left\{i_{v,w},j_{v,w}\right\}}$. Otherwise, i.e. *B*_*v *_∩ *B*_*w *_= *∅*, there are uniquely defined ≺-maximal base pairs {*k*_*v*_,*l*_*v*_} ∈ *B*_*v *_∖ *B*_*w*_ and {*k*_*w*_,*l*_*w*_} ∈ *B*_*w *_∖ *B*_*v*_ that enclose *v* and *w*, respectively. We note that *B*_*v *_∖ *B*_*w*_ (*B*_*w *_∖ *B*_*v*_) may be empty, in which case {*k*_*v*_,*l*_*v*_} ({*k*_*w*_,*l*_*w*_}) is also empty. This simple partition holds the key to computing distance distinguished partition functions below.

In the following, we assign the weights *a* for backbone edges and *b* for base pairs, respectively. Given a path *p*, we define the weight of the path *d*(*p*) as the sum of the weights of edges in the path. The (weighted) *graph-distance*$d_{v,w}^{G}$ in *G* is defined as the weight of the path *p* connecting *v* and *w* with *d*(*p*) being minimal. For the weights, we require the following condition: 

• If *i* and *j* are connected by an edge, then {*i*,*j*} ∈ *E* is the unique shortest path between *i* and *j*.

This condition ensures that single edges cannot be replaced by detours of shorter weight. Condition (W) and property (ii) of the secondary structure graphs implies *b *< 3*a* because the closing base pair must be shorter than a hairpin loop. Furthermore, considering a stacked pair we need *b *< *b *+ 2 *a*, i.e. *a *> 0. We allow the degenerate case *b *= 0 that neglects the traversals of base pairs.

Before we continue with the calculations of the partition function, let us first consider the problem formulation in more detail. For the FRET application, it is well-known that FRET efficiency is correlated with spatial distance. Furthermore, only a limited range of distance changes (e.g. 20 Å-100 Å for Cy3-Cy5) can be reported by the FRET experiments. Thus a more useful formulation of our problem is not to use the full expected quantity for all positions. Instead, we are interested in the average for all distance-values within some threshold *θ*_*d*_. As the space and time complexity will depend on the number of distances we consider, we will parametrise our complexity by the number of nucleotides *n* and the number of distances considered *D *= *θ*_*d *_+ 1, as well. In the worst case, there is *D *= *O *(*n*). However, given that in practice only a limited range of distance changes are considered, we rather view *D *= *O*(1) as a small constant in our contribution.

### Boltzmann distribution of graph-distances

For a fixed structure *G*, $d_{v,w}^{G}$ is easy to compute. Here, we are interested in the distribution $\text{Pr}[\;d_{v,w}^{G}|x]$ and its expected value $d_{v,w}=E[\;d_{v,w}^{G}|x]$ over the ensemble of all possible structures *G* for a given sequence *x*. Both quantities can be calculated from the Boltzmann distribution *Pr*[ *G*|*x*] = *e*^- *f *(*G*)/*R* *T*^/*Q* where $Q=\underset{G}{\sum }e^{-f(G)/\text{RT}}$ denotes the partition function of the ensemble of structures. As first shown in [15], *Q* and related quantities can be computed in quartic time. A reduction to a cubic algorithm may be obtained if the free energy of long interior loops may be regarded as prohibitive. This restriction has been widely used for long sequences [16]. Cubic runtime can also be achieved for some but not all parametrizations of interior loop energies [17].

A crucial quantity for our task is the restricted partition function

$$Z^{v,w}[\;d]=\underset{G\;\text{with}\;d_{v,w}^{G}=d}{\sum }e^{-f(G)/\text{RT}}$$

for a given pair *v*,*w* of positions in a given RNA sequence *x*. A simple computation (Appendix A in Additional file 1) verifies that the $\text{Pr}[\;d_{v,w}^{G}\;=\;d|x]=Z^{v,w}[\;d]/Q$ and $d_{v,w}=E[\;d_{v,w}^{G}|x]=\underset{d}{\sum }(Z^{v,w}[\;d]/Q)d$. Hence it suffices to compute *Z*^*v*,*w*^[*d*] for any 1 ≤ *d *≤ *n*. In the following sections we show that this can be achieved by a variant of McCaskill’s approach [15].

For the ease of presentation we describe in the following only the recursion for the simplified energy model for the “circular maximum matching”, in which energy contributions are associated with individual base pairs rather than loops. Our approach can be easily extended to the full model by using separating the partition functions into distinct cases for the loop types.

We use the letters *Z* and *Y* to denote partition functions with distance constraints, while *Q* is used for quantities that appear in McCaskill’s algorithm and are considered as pre-computed here. For instance, let $Q_{i,j}^{B}$ denote the partition function over all secondary structures on *x*[ *i*..*j*] that are enclosed by the base pair {*i*,*j*}. We will later also need the partition function *Q*_*i*,*j*_ over the sub-sequence *x *[ *i*..*j*], regardless of whether {*i*,*j*} is paired or not. In Additional file 1: Appendix C, we summarize the notations frequently used in our contribution.

### Recursions of *Z*^*v*,*w*^[ *d*]: The case when *v* and *w* are external

An important special case assumes that both *v* and *w* are external. This is the case e.g. when *v* and *w* are binded by proteins. In particular, the problem of computing end-to-end distances, i.e., *v *= 1 and *w *= *n*, is of this type.

Assuming (W), the shortest path between two external vertices *v*,*w* consists of the external vertices and their backbone connections together with the external base pairs. We call this path the *inside path* of *i*,*j* since it does not involve any vertices “outside” the subsequence *x *[ *i*..*j*].

For efficiently calculating the internal distance between any two vertices *v*,*w*, we denote by $Z_{i,j}^{I}[\;d]$ the partition function over all secondary structures on *x *[ *i*..*j*] with distance exactly *d*.

Now note that any structure on *x*[ *i*..*j*] starts either with an unpaired base or with a base pair connecting *i* to some position *k* satisfying *i *< *k *≤ *j*. In the first case, we have $d_{i,j}^{G}=d_{i,i+1}^{G}+d_{i+1,j}^{G}$ where $d_{i,i+1}^{G}=a$. In the second case, there exists $d_{i,j}^{G}=d_{i,k}^{G}+d_{k,k+1}^{G}+d_{k+1,j}^{G}$ with $d_{i,k}^{G}=b$ and $d_{k,k+1}^{G}=a$. Thus, $Z_{i,j}^{I}[\;d]$ can be split as follows, 

This gives the recursion

(1) $$Z_{i,j}^{I}[\;d]=Z_{i+1,j}^{I}[\;d-a]+\underset{i<k\leq j}{\sum }Q_{i,k}^{B}Z_{k+1,j}^{I}[d-b-a]$$

with the initialization $Z_{\text{ii}}^{I}[\;0]=1$ and $Z_{\text{ii}}^{I}[\;d]=0$ for *d *> 0. For consecutive vertices, we have $Z_{i,i+1}^{I}[\;a]=1$ and $Z_{i,i+1}^{I}[\;d]=0$ for *d *≠ *a*. These recursions have been derived in several different contexts, e.g. force induced RNA denaturations [6], the investigate of loop entropy dependence [9], the analysis of FRET signals in the presence of single-stranded binding proteins [5], as well as in mathematical studies of RNA panhandle-like structures [3,4].

In the following, it will be convenient to define also a special term for the empty structure. Setting $Z_{i,i-1}^{I}[\;-a]=1$ and $Z_{i,i-1}^{I}[\;d]=0$ for *d *≠ -*a* allows us to formally write an individual backbone edge as two edges flanking the empty structure and hence to avoid the explicit treatment of special cases. This definition of *Z*^*I*^ also includes the case that *i* and *j* are base paired in the recursion (1). This is covered by the case *k*=*j*, where we evaluate $Z_{j+1,j}^{I}[\;d-b-a]$. Since *d *= *b* is the only admissible value here, this refers to $Z_{j+1,j}^{I}[\;-a]$, which has the correct value of 1 due to our definition. Later on, we will also need *Z*^*I*^ under the additional condition that the path starts and ends with a backbone edge. We therefore introduce $Z^{I^{\prime }}$ defined as by

(2) $$Z_{i,j}^{I^{\prime }}[\;d]=Z_{i+1,j-1}^{I}[\;d-2a]$$

Note that if $Z_{i,j}^{I^{\prime }}[\;d]$ is called with *j *= *i *+ 1, then we call $Z_{i+1,i}^{I}[\;d-2a]$. The only admissible value again is the correct value *d*=*a*. In sum, we have the following 

This recursion requires *O*(*n*^3^*D*) time and *O*(*n*^2^*D*) space. It is possible to reduce the complexity of computing the expected distance in this special case by a linear factor. The trick is to use conditional probabilities for arcs starting at *i* or the conditional probability for *i* to be single-stranded, which can be determined from the partition function for RNA folding [3], see Additional file 1: Appendix B.

### Recursions of *Z*^*v*,*w*^[ *d*]: the general case

**Figure 1:**
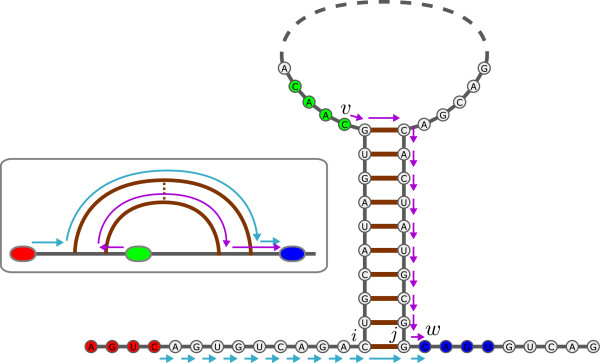
**Inside and outside paths.** The shortest path (violet arrows) from *v* (green) to *w* (blue) is not an inside path: *inside* emphasizes that, in contrast to the shortest path (cyan arrows) between the red region and *w*, it is not contained in the interval determined by its end points.

The distance between two positions *v* and *w* that are covered by an arc can be realized by both *inside paths* and *outside paths*. Here, “outside” emphasizes that the shortest path between two positions *v* and *w* contains vertex does not belongs to *x*[ *v*,*w*]. This case complicates the algorithmic approach, since both types of paths must be controlled simultaneously. Consider Figure 1, the shortest path between the green and blue regions includes some vertices outside the interval between these two regions. The basic idea is to generalize Equation (1) to computing the partition function *Z*^*v*,*w*^[ *d*]. The main question now becomes how to recurse over decompositions of both the inside and the outside paths.

Figure 1 shows that the outside paths are important for the green region, i.e., the region that is covered by an arc. Hence, we have to consider the different cases that the two positions *v* and *w* are covered by arcs. The set *Ω* of all secondary structures on *x* can be divided into two disjoint subclasses that have to be treated differently: 

• : *v* and *w* are not enclosed in a common base pair, i.e., *B*_*v *_∩ *B*_*w *_= *∅*.

• : there is a base pair enclosing both *v* and *w*, i.e., *B*_*v *_∩ *B*_*w *_≠ *∅*.

Note that this bipartition explicitly depends on *v* and *w*. In the following, we will first introduce the recursions that are required in *Ω*_0_ structures to compute *Z*^*v*,*w*^[ *d*].

#### Contribution of *Ω*_0_ structures to *Z*^*v*,*w*^[ *d*]: $Z_{0}^{v,w}[\;d]$

One example of this case is given in Figure 1 with the red and blue region, where *v* (vertex in green region) is covered by an arc, and *w* (vertex in blue region) is external. Denote the ≺-maximal base pair enclosing *v* by {*i*,*j*}. Since at most one of *v* and *w* is covered by an arc, we know that *j *< *w*. Hence, every path *p* from *v* to *w*, and hence also the shortest paths (not necessarily unique) must run through the right end *j* of the arc {*i*,*j*}. More precisely, there must sub-paths *p*_1_ and *p*_2_ with *d *(*p*) = *d*(*p*_1_) + *d *(*p*_2_) + *a* such that $v\overset{p}{⇝}w\to v\overset{p_{1}}{⇝}j-(j+1)\overset{p_{2}}{⇝}w$, where $i\overset{p}{⇝}j$ denotes that *p* is **a** shortest path from *i* to *j* and - denotes a single backbone edge. For the shortest path from *v* to *j*, it consists either of a shortest path $v\overset{p^{\prime }}{⇝}i$ and the arc {*i*,*j*}, or it goes directly to *j* without using the arc {*i*,*j*}.

How does this distinction translate to the partition function approach? If we want to calculate the contribution of this case to the partition function *Z*^*v*,*w*^[ *d*], we have to split both the sequence *x*[ *i*,*w*] and distance *d* as follows

a.) 

 where $Z_{j,w}^{I^{\prime }}[\;d_{2}]$ is the partition function starting and ending with a single-stranded base as defined in Equation (2), and $Z_{i,j}^{B,v}[\;d_{\ell },d_{r}]$ is the partition function consisting of all structures of *x*[ *i*,*j*] containing the base pair {*i*,*j*} with the property that the shortest path from *v* to *i* has length *d*_*ℓ*_ and the shortest path from *v* to *j* has length *d*_*r*_. In addition, *d*, *d*_*r*_ and *d*_2_ must satisfy *d *= *d*_*r *_+ *d*_2_.

The remaining cases for the contribution of the class *Ω*_0_ to *Z*^*v*,*w*^[ *d*] are given by all other possible combinations of *v* and *w* being single-stranded or being covered by an arc, i.e., 

To simplify, we extend the definition of $Z_{i,j}^{B,v}[\;d_{\ell },d_{r}]$ by setting $Z_{v,v}^{B,v}[\;0,0]=1$ and $Z_{v,v}^{B,v}[\;d_{\ell },d_{r}]=0$ for *d*_*ℓ *_+ *d*_*r *_> 0. This allows us to conveniently model all cases where either *v* or *w* are external, i.e., a.), b.), and d.), as special cases of c.).

In case c.), we have to split the distance *d* into five sub-distances $d_{l},d_{r},d_{l}^{\prime },d_{r}^{\prime },d_{I}$, in which *d*_*I*_ can be retrieved from the first four distances. Furthermore, we would require four splitting positions for the sequence for all possible combinations of *i*,*j*,*k*,*l*. A naïve implementation of this idea would result in an algorithm with time complexity *O*(*n*^6^*D*^5^) and space complexity *O*(*n*^2^*D*^2^).

A careful inspection shows, however, that the split of the distances for the arcs into *d*_*ℓ*_ and *d*_*r*_ is unnecessary. Since we want to know only distance to the left/right end, we can simply introduce two matrices $Z_{i,j}^{B,v,\ell }[\;d]$ and $Z_{i,j}^{B,v,r}[\;d]$ that store these values. These matrices can be generated from $Z_{i,j}^{B,v}[\;d_{\ell },d_{r}]$ as follows:

$$Z_{i,j}^{B,v,\ell }[\;d]=\underset{\begin{array}{c} d_{r}\\ d_{r}+b\geq d \end{array}}{\sum }Z_{i,j}^{B,v}[\;d,d_{r}]+\underset{\begin{array}{c} d_{\ell }\\ d_{\ell }>d \end{array}}{\sum }Z_{i,j}^{B,v}[\;d_{\ell },d-b]$$

Analogously, we compute $Z_{i,j}^{B,v,r}[\;d]$. In this way, we split the distance *d* into three contributions and we require four splitting positions for the sequence for all possible combinations of *i*,*j*,*k*,*ℓ*. 

Therefore, the contribution to *Z*^*v*,*w*^[ *d*] for structures in *Ω*^0^ is given by

(3) $$\begin{array}{l} \;Z_{0}^{v,w}[\;d]=\;\;\underset{\begin{array}{c} d_{1},d_{2}\\ d_{1}+d_{2}\leq d \end{array}}{\sum }\underset{\begin{array}{c} i,j,k,l\\ i\leq v\leq j<k\leq w\leq l \end{array}}{\sum }\;\;\left(\begin{array}{l} Q_{1,i-1}\cdot Z_{i,j}^{B,v,r}[\;d_{1}]\\ \cdot \;Z_{j,k}^{I^{\prime }}[\;d-(d_{1}+d_{2})]\\ \cdot \;Z_{k,l}^{B,w,\ell }[\;d_{2}]\cdot Q_{l+1,n} \end{array}\right) \end{array}$$

Note that for splitting the distance, we reuse the same indices (e.g., the *j* in $Z_{i,j}^{B,v,r}[\;d_{1}]\cdot Z_{j,k}^{I^{\prime }}[\;d-(d_{1}+d_{2})]$, where as for the remaining partition function, we use successive indices (e.g.,the *i* in $Q_{1,i-1}\cdot Z_{i,j}^{B,v,r}[\;d_{1}]$). This difference comes from the fact that splitting a sequence into subsequences is done naturally between two successive indices, whereas splitting a distance is naturally done by splitting at an individual position. We have only to guarantee that the substructures which participate in the split do agree on the structural context of the split position. This is guaranteed by requiring that $Z^{I^{\prime }}$ starts and ends with a backbone edge. We note that the incorporation of the full dangling end parameters makes is more tedious to handle the splitting positions.

This results in a complexity of *O*(*n*^6^*D*^3^) time and *O*(*n*^2^*D*) space. However, we do not need to split in *i*,*j*,*k*,*l* simultaneously. Instead, we could split case (c) at position *j* and introduce for all *v *≤ *j* and *k *≤ *w* the auxiliary variables

$$\begin{array}{ll} Z_{1,j}^{B,v,r}[\;d_{1}] & =\underset{i\leq v}{\sum }Q_{1,i-1}\cdot Z_{i,j}^{B,v,r}[\;d_{1}]\\ Z_{k,n}^{B,w,\ell }[\;d_{2}] & =\underset{w\leq l}{\sum }Z_{k,l}^{B,w,\ell }[\;d_{2}]\cdot Q_{l+1,n}\\ Z_{j,n}^{I\;B,w,\ell }[\;d^{\prime }] & =\underset{k>j}{\sum }\underset{\begin{array}{c} d_{2}\leq d^{\prime } \end{array}}{\sum }Z_{j,k}^{I^{\prime }}[\;d^{\prime }-d_{2}]\cdot Z_{k,n}^{B,w,\ell }[\;d_{2}]. \end{array}$$

Finally, we can replace recursion (3) by

(4) $$Z_{0}^{v,w}[\;d]=\underset{v\leq \;j}{\sum }\underset{d_{1}\leq d}{\sum }Z_{1,j}^{B,v,r}[\;d_{1}]\cdot Z_{j,n}^{I\;B,w,\ell }[\;d-d_{1}]$$

We thus arrive at *O*(*n*^3^*D*^2^) time and *O*(*n*^2^*D*) space complexity for the contribution of *Ω*_0_ structures to *Z*^*v*,*w*^[ *d*], excluding the complexity of computing $Z_{i,j}^{B,v}[\;d_{\ell },d_{r}]$.

#### Contribution of *Ω*_1_ structures to *Z*^*v*,*w*^[ *d*]

*Ω*_1_ contains all cases where *v* and *w* are covered by a base pair. In the following, let {*p*,*q*} be the ≺-minimal base pair covering *v* and *w*. In principle, this case looks similar to the case for *Ω*_0_. However, we have to take into considerations the paths between *v* and *w* over the base pair {*p*,*q*}. Thus, we need to store the partition function for all inside and outside for each ≺-minimal arc {*p*,*q*} that covers *v* and *w*, which we will call $Z_{p,q}^{v,w}[\;d_{O},d_{I}]$. In principle, a similar recursion as defined for $Z_{0}^{v,w}$ in equation (3) can be derived, with the additional complication since we have to take care of the additional outside distance due to the arc {*p*,*q*}. Thus, we obtain the following splitting: 

Again we can avoid the complexity of simultaneously splitting at {*i*,*j*}*and* {*k*,*l*} by doing a major split after *j*. Thus, we get the following picture, 

 which leads to the following equivalent recursions:

(5) $$Y_{p,j}^{B,v,r}[\;d,d_{r}]=\underset{p<i\leq v}{\sum }\underset{d_{O}^{\prime }\leq d}{\sum }Z_{p,i}^{I^{\prime }}[\;d_{O}^{\prime }]\cdot Z_{i,j}^{B,v}[\;d-d_{O}^{\prime },d_{r}]$$

(6) $$Y_{k,q}^{B,w,\ell }[\;d_{\ell }^{\prime },d^{\prime }]=\underset{w\leq l<q}{\sum }\underset{d_{O}^{\prime \prime }\leq d^{\prime }}{\sum }Z_{k,l}^{B,w}[\;d_{\ell }^{\prime },d^{\prime }-d_{O}^{\prime \prime }]\cdot Z_{l,q}^{I^{\prime }}[\;d_{O}^{\prime \prime }]$$

(7) $$\;Y_{j,q}^{I\;B,w,\ell }[\;d_{\text{I}\ell },d^{\prime }]=\underset{j<k<q}{\sum }\underset{\begin{array}{c} d_{\ell }^{\prime }\leq d_{\text{I}\ell } \end{array}}{\sum }Z_{j,k}^{I^{\prime }}[\;d_{\text{I}\ell }-d_{\ell }^{\prime }]\cdot Y_{k,q}^{B,w,\ell }[\;d_{\ell }^{\prime },d^{\prime }]$$

Overall, we get the following recursion:

(8) $$\;Z_{p,q}^{v,w}[\;d_{O},d_{I}]=\underset{v\leq \;j}{\sum }\underset{\begin{array}{c} d_{r}\leq d_{I}\\ d\leq d_{O} \end{array}}{\sum }Y_{p,j}^{B,v,r}[\;d,d_{r}]\cdot Y_{j,q}^{I\;B,w,\ell }[\;d_{I}-d_{r},d_{O}-d]$$

We can now define *Z*^*v*,*w*^[ *d*] by

(9) $$\;\begin{array}{ll} Z^{v,w}[\;d]=Z_{0}^{v,w}[\;d]+{\hat{Q}}_{p,q}^{b}\cdot & \left\{\underset{\begin{array}{c} \left\{p,q\}\neq \{v,w\right\}\\ d_{I}\geq d+b \end{array}}{\sum }Z_{p,q}^{v,w}[\;d,d_{I}]+\underset{\begin{array}{c} \left\{p,q\}\neq \{v,w\right\}\\ d<d_{O}+b \end{array}}{\sum }Z_{p,q}^{v,w}[\;d_{O},d]\right\} \end{array}$$

where ${\hat{Q}}_{p,q}^{b}$ is the external partition function over all structures on the union of the intervals *x*[ 1..*p*] ∪ *x*[ *q*..*n*] so that {*p*,*q*} is a base pair. Since the base pair probability can be written as $\text{Pr}(\{p,q\})=\frac{{\hat{Q}}_{p,q}^{b}Q_{p,q}^{b}}{Q}$, this quantity can be calculated as ${\hat{Q}}_{p,q}^{b}=\frac{\text{Pr}(\{p,q\})Q}{Q_{p,q}^{b}}$. The base pair probability *Pr *({*p*,*q*}), and the partition functions *Q* and $Q_{p,q}^{b}$ are computed by means of McCaskill’s algorithm.

This part now has a complexity of *O*(*n*^2^*D*^2^) space and *O*(*n*^3^*D*^4^) time. For practical applications, however, we do not need to consider all possible {*p*,*q*}. Instead, there are only few base pairs that are likely to form *and* that cover *v*,*w*, especially for *v*,*w* where the internal distance of *v*,*w* is large enough such that an outside path has to be considered at all. If we assume a constant number of such long-range base-pairs, then the complexity is reduced by an *n*^2^-factor. For the complexity in terms of distance, recall that *D* is typically small.

### Recursions for $Z_{i,j}^{B,v}[d_{\ell },d_{r}]$

So far, we have used $Z_{i,j}^{B,v}[\;d_{\ell },d_{r}]$ as a black box. In order to compute these terms, we distinguish the limiting cases a.) *v *= *i*, b.) *v *= *j*, c.) is external from the generic case d.): 

Starting from the limiting cases, we initialize $Z_{v,j}^{B,v}[\;0,d_{r}]$ as follows:

$$Z_{v,j}^{B,v}[\;0,d_{r}]=\left\{\begin{array}{ll} Z_{v,j}^{I^{\prime }}[\;d_{r}] & \;\text{for}\;a\leq d_{r}<b\\ \underset{d^{\prime }\geq b}{\sum }Z_{v,j}^{I^{\prime }}[\;d^{\prime }] & \;\text{for}\;d_{r}=b\\ 0 & \text{otherwise} \end{array}\right)$$

and analogously for $Z_{i,v}^{B,v}[\;d_{\ell },0]$. Furthermore, $Z_{i,j}^{B,v}[\;0,0]=0$ for *i *≠ *v *≠ *j*. These conventions allow us to model all cases as special cases of *d*). Our key observation here is that the dependency between *d*_*ℓ*_ and *d*_*r*_ can be used to reduce the time complexity. Instead of using the variables *d*_*ℓ*_ and *d*_*r*_ in $Z_{i,j}^{B,v}[\;d_{\ell },d_{r}]$, we use the pair *d*_*ℓ*_,*d*_add_ in $Z_{i,j}^{B,v}[\;d_{\ell },d_{\ell }+d_{\text{add}}]$. Similarly, we use $d_{\ell }^{\prime },d_{\text{add}}^{\prime }$ instead of $d_{\ell }^{\prime },d_{r}^{\prime }$ for the inner base pair, which then determines completely the splitting the distances. This results in an recursion for $Z_{i,j}^{B,v}[\;d_{\ell },d_{\ell }+d_{\text{add}}]$ with complexity $O(n^{4}D^{2}c_{b}^{2})$ time and *O *(*n*^2^*Dc*_*b*_) space. To be precise, there are three sub-cases as follows. 

The values that are chosen to split *d*_*ℓ*_ and *d*_add_ are indicated in green and blue. When the arc {*i*,*j*} is colored violet, then there is a shortest path that does not use the distance marked in red but uses the other direction together with the arc {*i*,*j*}. If -*b*<*d*_*add*_<+*b*, then we know that neither a shortest path $v\overset{p}{⇝}i$ nor $v\overset{p}{⇝}j$ uses the arc {*i*,*j*}. The left distance is thus given by $d_{\ell }-d_{\ell }^{\prime }$. Using the shortcuts *d*_*r *_= *d*_*ℓ *_+ *d*_add_ and $d_{r}^{\prime }=d_{\ell }^{\prime }+d_{\text{add}}^{\prime }$, then the distance between *l* and *j* must be $d_{r}-d_{r}^{\prime }=(d_{\ell }+d_{\text{add}})-\left(d_{\ell }^{\prime }+d_{\text{add}}^{\prime }\right)$. If, on the other hand, *d*_add _= + *b*, then we know that there is at least one shortest path that can be composed by using a shortest path v⇝i, followed by the arc {*i*,*j*}. This of course implies that the shortest path $v\overset{p}{⇝}j$ is has exactly the length *d*_*ℓ *_+ *b*, or is larger. For a sub-path $l+1\overset{p^{\prime }}{⇝}j$ this implies that the length is greater or equal $d=d_{r}-d_{r}^{\prime }=\left(d_{\ell }+b\right)-\left(d_{\ell }^{\prime }+d_{\text{add}}^{\prime }\right)$. Thus, we just have to add all partition functions $Z_{k,j}^{I^{\prime }}[\;d^{\prime }]$ with *d*^′ ^> *d*. This can be done efficiently by using a precalculated matrix $Z_{i,j}^{I^{\prime }\geq }[\;d]$, which is defined as $\underset{d^{\prime }\geq d}{\sum }Z_{i,j}^{I^{\prime }}[\;d^{\prime }]$. Note that $Z_{i,j}^{I^{\prime }\geq }[\;d]$ can also be defined if we restrict in all recursion the distance *d* to a threshold *θ*_*d*_, since $Z_{i,j}^{I^{\prime }\geq }[\;d]=\underset{d^{\prime }\geq d}{\sum }Z_{i,j}^{I^{\prime }}[\;d^{\prime }]=Q_{i,j}^{\prime }-\underset{d^{\prime }<d}{\sum }Z_{i,j}^{I^{\prime }}[\;d^{\prime }]$. In which, where $Q_{i,j}^{\prime }$ is *Q*_*i *+ 1,*j *- 1_ if *j *> *i *+ 1, 1 if *j *= *i *+ 1 and 0 otherwise. Note, furthermore, that all $Z_{i,j}^{I^{\prime }}[\;d^{\prime }]$ for *d*^′^< *d *≤ *θ*_*d*_ are calculated when we restrict the distance to *θ*_*d*_.

Finally, if *d*_ad d_= - *b*, then the shortest path $l\overset{p}{⇝}j$ has distance $(d_{\ell }-b)-\left(d_{\ell }^{\prime }+d_{\text{add}}^{\prime }\right)$. For the shortest path $k\overset{p}{⇝}i$, we know that it has length $d_{\ell }-d_{\ell }^{\prime }$ or greater, which can be resolved by again using $Z_{i,k-1}^{I^{\prime }\geq }[\;d_{\ell }-d_{\ell }^{\prime }]$. Thus, we get the following optimized recursion for $Z_{i,j}^{B,v}[\;d_{\ell },d_{\ell }+d_{\text{add}}]$ with *d*_*ℓ *_≠ 0 and *d*_*ℓ *_+ *d*_add _≠ 0:

(10) $$Z_{i,j}^{B,v}\left[d_{l},d_{l}+d_{\text{add}}\right]=\left\{\begin{array}{l} \underset{\underset{\underset{v\leq l<j}{i<k\leq v}}{k\neq l}}{\sum }\underset{d_{l}^{\prime }\leq d_{l}}{\sum }\underset{\underset{-b\leq d_{\text{add}}^{\prime }\leq b}{d_{\text{add}}^{\prime }}}{\sum }\left(\begin{array}{l} Z_{i,k}^{I^{\prime }}\left[d_{l}-d_{l}^{\prime }\right]\cdot Z_{k,l}^{B,v}\left[d_{l}^{\prime },d_{l}^{\prime }+d_{\text{add}}^{\prime }\right]\\ \cdot Z_{l,j}^{I^{\prime }}\left[\left(d_{l}+d_{\text{add}}\right)-\left(d_{l}^{\prime }+d_{\text{add}}^{\prime }\right)\right] \end{array}\right)\;\text{if}\;-b<d_{\text{add}}<b\\ \underset{\underset{\underset{v\leq l<j}{i<k\leq v}}{k\neq l}}{\sum }\underset{d_{l}^{\prime }\leq d_{l}}{\sum }\underset{\underset{-b\leq d_{\text{add}}^{\prime }\leq b}{d_{\text{add}}^{\prime }}}{\sum }\left(\begin{array}{l} Z_{i,k}^{I^{\prime }}\left[d_{l}-d_{l}^{\prime }\right]\cdot Z_{k,l}^{B,v}\left[d_{l}^{\prime },d_{l}^{\prime }+d_{\text{add}}^{\prime }\right]\\ \cdot Z_{l,j}^{I^{\prime }}\geq \left[\left(d_{l}-b\right)-\left(d_{l}^{\prime }+d_{\text{add}}^{\prime }\right)\right] \end{array}\right)\;\text{if}\;d_{\text{add}}=b\\ \underset{\underset{\underset{v\leq l<j}{i<k\leq v}}{k\neq l}}{\sum }\underset{d_{l}^{\prime }\leq d_{l}}{\sum }\underset{\underset{-b\leq d_{\text{add}}^{\prime }\leq b}{d_{\text{add}}^{\prime }}}{\sum }\left(\begin{array}{l} Z_{i,k}^{I^{\prime }}\left[d_{l}-d_{l}^{\prime }\right]\cdot Z_{k,l}^{B,v}\left[d_{l}^{\prime },d_{l}^{\prime }+d_{\text{add}}^{\prime }\right]\\ \cdot Z_{l,j}^{I^{\prime }}\left[\left(d_{l}-b\right)-\left(d_{l}^{\prime }+d_{\text{add}}^{\prime }\right)\right] \end{array}\right)\;\text{if}\;d_{\text{add}}=-b \end{array}\right)$$

## Discussion and applications

The theoretical analysis of the distance distribution problem shows that, while polynomial-time algorithms exist, they probably cannot be improved to space and time complexities that make them widely applicable to large RNA molecules. Due to the unfavorable time complexity of the current algorithm and the associated exact implementation in C, a rather simple and efficient sampling algorithm has been implemented. We resort to sampling Boltzmann-weighted secondary structures with RNAsubopt -p[16], which uses the same stochastic backtracing approach as sfold[18]. As the graph-distance for a pair of nucleotides in a given secondary structure can be computed in *O*(*n* log*n*) time by Dijkstra’s algorithm with Fibonacci heap [19], even large samples can be evaluated efficiently.

**Figure 2:**
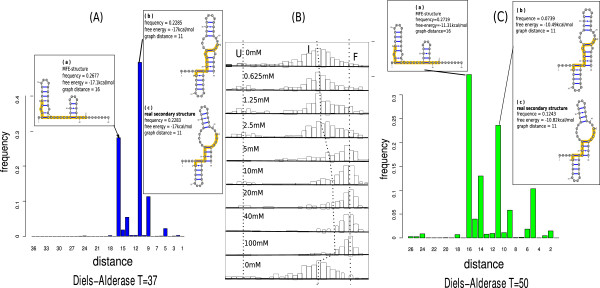
**Relation between graph-distance distribution and smFRET data. (A)** The graph-distance distribution of a Diels-Alderase (DAse) ribozyme at temperature 37°C. Structures **(a)**, **(b)** and **(c)** are the top three secondary structures considering their free energy: the minimum free energy structure is shown in **(a)**, **(c)** is the experimentally determined secondary structure, which is ranked as the 3rd best sub-optimal structure with RNAsubopt -e. The graphic representations of these structures are produced with VARNA[20]. **(B)** The corresponding smFRET efficiency (*E*_*fret*_) histograms are reported in [12]. From these data, three separate states of the DAse ribozyme can be distinguished, the unfolded (U), intermediate **(I)** and folded **(F)** states. **(C)** The graph-distance distribution in the ensemble which is approximated with RNAsubopt -p at temperature 50°C.

As we pointed out in the introduction, the graph-distance measure introduced in this paper can serve as a first step towards a structural interpretation of smFRET data. As an example, we consider the graph distance distribution of a Diels-Alderase (DAse) ribozyme (Figure 2A). Histograms of smFRET efficiency (*E*_*fret*_) for this 49 nt long catalytic RNA are reported in [12] for a large number of surface-immobilized ribozyme molecules as a function of the Mg ^2+^ concentration in the buffer solution. A sketch of their histograms is displayed in Figure 2B. The dyes are attached to sequence positions 6 (Cy3) and 42 (Cy5) and hence do not simply reflect the end-to-end distance, Figure 2A(c). In this example, we observe the expected correspondence small graph-distances with a strong smFRET signal. This is a particular interesting example, since the minimal free energy (mfe) structure (Figure 2A(a)) predicted with RNAfold is not identified with the real secondary structure (Figure 2A(c)). In fact, the ground state secondary structure is ranked as the 3rd best sub-optimal structure derived via RNAsubopt -e. The free energy difference between these two structures is only 0.1 kcal/mol. However, their graph-distances show a relatively larger difference. The 2nd best sub-optimal structure (Figure 2A(b)) looks rather similar with the 3rd structure, in particular, they share the same graph-distance value.

The smFRET data of [12] indicates the presence of three sub-populations, corresponding to three different structural states: folded molecules (state F), intermediate conformation (state I) and unfolded molecules (state U). In the absence of Mg ^2+^, the I state dominates, and only small fractions are found in states U and F. Unfortunately, the salt dependence of RNA folding is complex [21,22] and currently is not properly modeled in the available folding programs. We can, however, make use of the qualitative correspondence of low salt concentrations with high temperature. In Figure 2C we therefore re-compute the graph-distance distribution in the ensemble at an elevated temperature of 50°C. Here, the real structure becomes the second best structure with free energy -10.82 kcal/mol and we observe a much larger fraction of (nearly) unfolded structures with longer distances between the two beacon positions. Qualitatively, this matches the smFRET data showed in Figure 2B.

**Figure 3:**
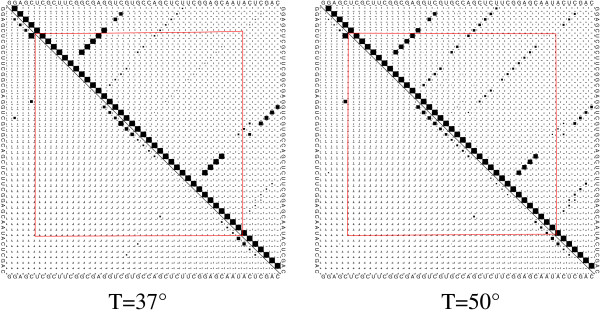
**Comparison between the base-pair probabilities and the distance importance *I***_**6,42**_**(*e*).** The base-pair probabilities (upper-right-triangle) and the distance importances *I*_6,42_(*e*) (lower-left-triangle) of backbone edges and base pairs between 6(U) and 42(U) of DAse ribozyme (Figure 2) are computed at temperatures 37°C and 50°C, repectively. The size of the squares is proportional to the probability/value. The region covered by the between 6(U) and 42(U) is annotated by a red rectangle. For ease of comparison, backbone edges are added to the base-pair probability matrix.

Furthermore, for a given pair *v*,*w* of positions in a given RNA sequence *x*, the importance *I*_*v*,*w*_(*e*) of a backbone edge or base pair *e* in calculating the graph-distance distribution is evaluated by $I_{v,w}(e)=\underset{e\in \Pi_{e}}{\sum }\text{Pr}[\;G|x]$, where the set *Π*_*e*_ comprises the secondary structures *G* with (at least) one shortest path between *v* and *w* that runs through *e*. Figure 3 compares dot plots of *I*_*v*,*w*_(*e*) with the base-pair probabilities in the RNA structure ensemble of the DAse ribozyme at temperatures 37°C and 50°C. Since RNAgraphdist computes only one of possible many shortest paths for each *G*, hence we obtain only a lower bound on *I*_*v*,*w*_(*e*).

We observe for DAse that the contributions from the backbone edges are larger than the base pairs at both temperatures. For *T *= 37°C, there are in total 14 edges with *I*_6,42_(*e*) > 0.4. Only two of them, 5(C)–18(G) and 2(G) – 21(C) are base pairs. For *T *= 50°C, there is only the pair 5(C) – 18(G) is heavily used (*I*_6,42_(5,18) = 0.636). Combining the analysis of data illustrated in Figure 2, it may indicate that the existences of two base pairs, 2(G) – 21(C) and 28(G) – 39(C) can affect the graph-distance distribution of RNA secondary structure ensemble and consequently affect smFRET measurements. Such constraints may become an interesting source of constraints for RNA structure prediction.

**Figure 4:**
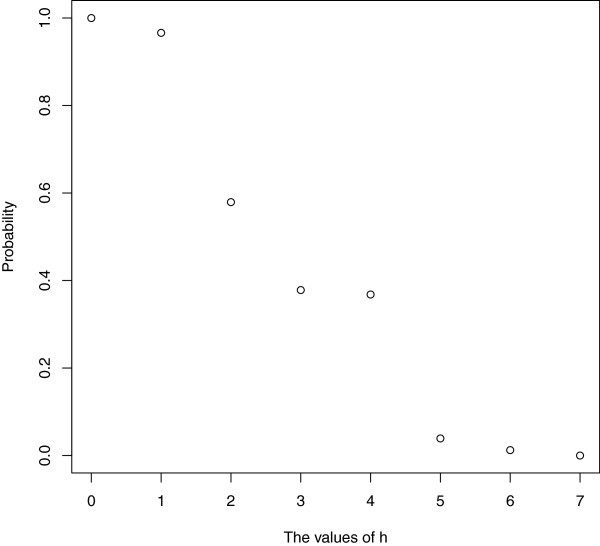
**“Outside-path” distribution of DAse ribozyme.** The distribution of paths which pass through positions outside the sequence interval *x*[6-*h*,42+*h*] of DAse ribozyme (Figure 2). As expected, this probability drops fast to 0 with respect to *h*.

In addition, we compute the distribution of paths which pass through positions outside sequence interval *x*[ 6-*h*,42+*h*] of DAse ribozyme. As illustrated in Figure 4, this “outside-path” distribution, as expected, drops fast to 0 with respect to *h*.

**Figure 5:**
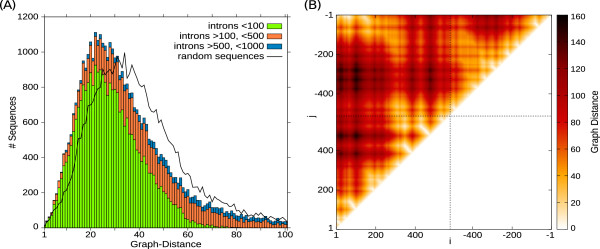
**Graph-distance distribution of the *Drosophila melanogaster *and the genomic RNA of human *Coronavirus*229E. (A)**: Distribution of graph-distances (*a *= *b *= 1) in *Drosophila melanogaster* pre-mRNAs between the first and last intron position. To save computational resources, pre-mRNAs were truncated to 100 nt flanking sequence of introns. The black curve shows the graph-distance distribution computed for the corresponding pairs of positions on sequences that were randomized by di-nucleotide shuffling. **(B)**: Graph-distances (*a *= *b *= 1) within and between the 5’ and 3’ regions of the genomic RNA of human *Coronavirus* 229E computed from a concatenation of position 1–576 (5’ UTR) and 25188–25688 (upstream of gene N). Secondary structures bring the 5’ TRS-L (63–76) and 3’ TRS-B (-23– -10) elements into close proximity.

Long-range interactions play an important role in pre-mRNA splicing and in the regulation of alternative splicing [23-25], bringing splice donor, acceptor, branching site into close spatial proximity. Figure 5A shows for *D. melanogaster* pre-mRNAs that the distribution of graph-distances between donor and acceptor sites shifted towards smaller values compared to randomly selected pairs of positions with the same distance. Due to the insufficiency of the spacial-distance information of structural elements in the secondary structures, we artificially choose *a *= *b *= 1 in our experiments. Although the effect is small, it shows a clear difference between the real RNA sequences and artificial sequences that were randomized by di-nucleotide shuffling. Furthermore, Table 1 displays for a specific intron CG16979-RA_intron_0_0_chr3L_15569803 from *Drosophila melanogaster* (dm3), the most probable secondary structures in the sub-ensembles of secondary structures such that their graph-distances are 7, 6, and 14, respectively.

**Table 1:** **Graph-distance of intron CG16979-RA_intron_0_0_chr3L_15569803 from *Drosophila melanogaster *** **(dm3)**

**1st**	**6th**	**10th**
**Distance = 7**	**Distance = 6**	**Distance = 14**
a	b	c

The intron is extended at the 5’ and 3’ end with 100 bases. The graph-distance is computed between *i *= 101(G) and *j*=159(G) (annotated in the figure). The corresponding shortest paths are highlighted in yellow. The structures (a), (b) and (c) are the most stable structures considering the sub-ensembles which are the sets of structures of graph-distance 7, 6 and 14, respectively. The graph distances 7, 6 and 14 are the 1st, 6th and 10th most favourable graph-distances considering Boltzmann facor.

**Figure 6:**
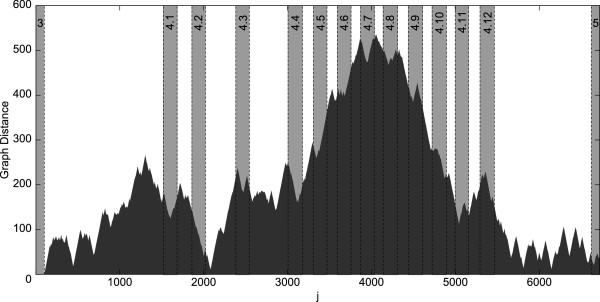
**Graph-distance distribution of DSCAM.** Graph-distance distribution of DSCAM from last nucleotide of exon 3 (Chr.2, Pos. 3255892) to any position until exon 5 (Chr.2, Pos. 3249372), including all 12 variations of alternative exon 4. For secondary structure prediction 100 nt flanking region were used.

The *Drosophila melanogaster* Down syndrome cell adhesion molecules (DSCAM) encodes for 38.016 different mRNAs by alternative splicing. Among the 24 exons, exon 4 alone has 12 variants [26]. In Figure 6 we display the graph-distance from donor (exon 3) to any downstream position until acceptor (exon 5). Comparing the graph-distances of all twelve acceptors of exon 4, we see clearly local peaks. This suggests the acceptor being part of hairpin loops, three dimensionally poking out of the long transcript to interact easily with the spliceosome and donor. Four of the twelve acceptor sites show no local peak, however seem to be accessible as internal loops of longer hairpins.

The spatial organization of the genomic and sub-genomic RNAs is important for the processing and functioning of many RNA viruses. This goes far beyond the well-known panhandle structures. In *Coronavirus* the interactions of the 5’ TRS-L cis-acting element with body TRS elements has been proposed as an important determinant for the correct assembly of the *Coronavirus* genes in the host [27]. The mechanisms of interaction is unknown, and a small three-dimensional distance is suspected. The matrix of expected graph-distances in Figure 5B shows that TRS-L and TRS-B are indeed placed close to each other. In Table 2, we show the most stable structures within the sub-ensembles of secondary structures such that their graph-distances are 14, 5, and 35, respectively. All these RNA secondary structures brings the leader transcription regulation site (L-TRS) in close spatial proximity with the body transcription regulation site (B-TRS).

**Table 2:** **Graph-distance of the genomic RNA of** ***human Coronavirus 229E* computed from a concatenation of position 1-576 and 25188-25688**

**1st**	**6th**	**8th**
**Distance = 14**	**Distance = 5**	**Distance = 35**
a	b	c

The graph-distance is measured from the most 5’ end to the most 3’ end of the sequence. The RNA secondary structure brings the leader transcription regulation site (L-TRS) in close spatial proximity with the body transcription regulation site (B-TRS). The structures (a), (b) and (c) are the most stable structures considering the sub-ensembles which are the sets of structures of graph-distance 14, 5 and 35, respectively. These are the 1st, 6th and 8th most favoured graph-distances in the Boltzmann ensemble.

These examples indicate that the systematic analysis of the graph-distance distribution both for individual RNAs and their aggregation over ensembles of structures can provide useful insights into structural influences on RNA function. These may not be obvious directly from the structures due to the inherent difficulties of predicting long-range base pairs with sufficient accuracy and the many issues inherent in comparing RNA structures of very disparate lengths.

Due the complexity of algorithm we have refrained from attempting a direct implementation in an imperative programming language. Instead, we are aiming at an implementation in Haskell that allows us to make use of the framework of algebraic dynamic programming [28]. The graph-distance measure and the associated algorithm can be extended in principle to of RNA secondary structures with additional tertiary structural elements such as pseudoknots [29] and G-quadruples [30]. RNA-RNA interaction structures [31] also form a promising area for future extensions. We note finally, that the Fourier transition method introduced in [32] could be employed to achieve a further speedup.

## Conclusion

The distribution of spatial distances in the equilibrium structure ensemble of an RNA molecule carries information about the overall structure of the molecule. These distance can be approximated by the graph-distance in RNA secondary structure. We introduced a polynomial time algorithm to compute the equilibrium distribution of graph-distances between a fixed pair of nucleotides. For practical applications, small distances are of main interest. Here, the time complexity of the proposed algorithm is *O*(*n*^4^), compared to a naïve implementation with time complexity of *O*(*n*^11^) for sequence length *n* and distances that can cover the whole sequence length. Since further reductions, however, seem to be difficult, we also introduced sampling approaches that are much easier to implement. They are also theoretically favorable for several real-life applications, in particular since these primarily concern long-range interactions in very large RNA molecules.

## Competing interests

The authors declare that they have no competing interests.

## Authors’ contributions

Conceived and designed the algorithms: JQ, PFS and RB. Implemented algorithms and performed experiments: JQ and MN. Analyzed Diels-Alderase ribozyme data: JQ and PFS. Analyzed pre-mRNA splicing data: MN and MM. Wrote the final manuscript: JQ, MM, PF and RB. All authors read and approved the final manuscript.

## Supplementary Material

Additional file 1 **Appendix A: Proof of the **$E[\;d_{G}(v,w)]=\underset{d}{\sum }d\times$$\frac{Z^{v,w}[\;d]}{Z}$**.** Appendix B: The conditional probability for *i* to be single-stranded can be determined from the partition function for RNA folding. Appendix C: Tables of notations. Click here for file
