# Supplementary material for: Graph-distance distribution of the Boltzmann ensemble of RNA secondary structures
Source: Algorithms Mol Biol. 2014 Sep 11;9:19. doi: 10.1186/1748-7188-9-19 (PMC4181469; doi:10.1186/1748-7188-9-19)
Supplement: Additional file 1 — Appendix A: Proof of the E[dG(v,w)]=∑dd×Zv,w[d]Z. Appendix B: The conditional probability for i to be single-stranded can be determined from the partition function for RNA folding. Appendix C: Tables of notations. [file 1748-7188-9-19-S1.pdf]

Additional File 1: GRAPH-DISTANCE DISTRIBUTION OF THE BOLTZMANN ENSEMBLE OF RNA SECONDARY STRUCTURES

April 27, 2014

1 Appendix A: Proof of the $E[d_G(v,w)] = \sum_d d \times \frac{Z^{v,w}[d]}{Z}$

Proof: $E[d_G(v,w)] = \sum_d d \times \frac{Z^{v,w}[d]}{Z}$

$$\begin{aligned} E[d_G(v,w)] &= \sum_G d_G(v,w) \times Pr[G|\xi] = \sum_d \sum_{G \text{ with } d_G(v,w)=d} d \times \frac{e^{-f(G)/RT}}{Z} \\ &= \sum_d d \times \frac{\sum_{G \text{ with } d_G(v,w)=d} e^{-f(G)/RT}}{Z} = \sum_d d \times \frac{Z^{v,w}[d]}{Z} \end{aligned}$$

2 Appendix B: The conditional probability for i to be single-stranded can be determined from the partition function for RNA folding.

Theorem 2.1 *The expected distance $E[d^G_{i,j}]$ can be calculated as:*

$$E[d^G_{i,j}] = (a + E[d^G_{i+1,j}]) \cdot \frac{1 \cdot Q_{i+1,j}}{Q_{i,j}} + \sum_{i<k\leq j} (b + E[d^G_{k+1,j}]) \cdot \frac{Q^b_{i,k} \cdot Q_{k+1,j}}{Q_{i,j}} \quad (1)$$

Let G be a structure. For simplicity of notation, we write $G = \bullet G'$ if the first position is unpaired, and $G = (\ldots)_j G'$ is the first base is paired to some position j, and G' is the substructure of G starting from position $j+1$. Alternatively, we may use the notation $(i,j) \in G$ for the case where the position i and j are base paired in G.

The expected length $E[d_G(i,j)]$ can be calculated as: follows:

$$\begin{aligned} E[d_G(i,j)] &= \sum_{G \text{ struct. of } \xi[i\ldots j]} d_G(i,j) Pr[G|\xi[i\ldots j]] \\ &= \sum_{G=\bullet G'} (a + d_{G'}(i,j)) Pr[G|\xi[i\ldots j]] \quad + \sum_{i<k\leq j} \sum_{G=(\ldots)_k G'} (b + d_{G'}(i,j)) Pr[G|\xi[i\ldots j]] \\ &\overset{def.}{=} \qquad EL_{sg} \qquad + \sum_{i<k<j} \qquad EL_{bp(k)} \end{aligned}$$

Now EL_{sg} can be simplified as follows:

$$\begin{aligned} EL_{sg} &= \sum_{G=\bullet G'} (a + d_{G'}(i,j)) Pr[G|\xi[i\ldots j]] \\ &= \left(\sum_{G=\bullet G'} a \cdot Pr[G|\xi[i\ldots j]] \right) + \left(\sum_{G=\bullet G'} d_{G'}(i,j) \cdot Pr[G|\xi[i\ldots j]] \right) \\ &= a \cdot Pr[G = \bullet G'|\xi[i\ldots j]] + \left(\sum_{G=\bullet G'} d_{G'}(i,j) \cdot Pr[G|\xi[i\ldots j]] \right), \end{aligned}$$

where $Pr[G = \bullet G'|\xi[i\ldots j]]$ can be calculated as the probability of the first position to be single-stranded in the sequence $\xi[i\ldots j]$, i.e.,

$$Pr[G = \bullet G'|\xi[i\ldots j]] = \frac{1 \cdot Q_{i+1,j}}{Q_{i,j}}$$

We are also able to push the second term since

$$\sum_{G=\bullet G'} d_{G'}(i,j) \cdot Pr[G|\xi[i\ldots j]] = \sum_{G'} d_{G'}(i,j) \cdot Pr[\bullet G'|\xi[i\ldots j]]$$

Now we know that for every G' we have that the Boltzmann weighted energy of G' is part of the partition function of $Q_{i+1,j}$. Thus we get

$$
\begin{aligned}
&= \sum_{G'} d_{G'}(i,j) \cdot \frac{\exp(-E(\bullet G')/kT)}{Q_{i,j}} \\
&= \sum_{G'} d_{G'}(i,j) \cdot \frac{\exp(-E(G')/kT)}{Q_{i+1,j}} \frac{Q_{i+1,j}}{Q_{i,j}} \\
&= \frac{Q_{i+1,j}}{Q_{i,j}} \sum_{G'} d_{G'}(i,j) \cdot \frac{\exp(-E(G')/kT)}{Q_{i+1,j}} \\
&= Pr[G = \bullet G' | \xi[i \dots j]] \sum_{G'} d_{G'}(i,j) \cdot Pr[G' | \xi[i+1 \dots j]] \\
&= Pr[G = \bullet G' | \xi[i \dots j]] \cdot E[d_G(i+1,j)]
\end{aligned}
$$

Overall we get

$$EL_{sg} = (a + E[d_G(i+1,j)]) \cdot Pr[G = \bullet G' | \xi[i \dots j]]$$

For the term $EL_{bp(k)}$, we have a similar reduction:

$$
\begin{aligned}
EL_{bp(k)} &= \sum_{G=(\dots)_k G'} (b + d_{G'}(i,j)) Pr[G | \xi[i \dots j]] \\
&= \left(\sum_{G=(\dots)_k G'} b \cdot Pr[G | \xi[i \dots j]] \right) + \left(\sum_{G=(\dots)_k G'} d_{G'}(i,j) Pr[G | \xi[i \dots j]] \right) \\
&= (b \cdot Pr[G = (\dots)_k G' | \xi[i \dots j]]) + \left(\sum_{G=(\dots)_k G'} d_{G'}(i,j) Pr[G | \xi[i \dots j]] \right),
\end{aligned}
$$

where $Pr[G = (\dots)_k G' | \xi[i \dots j]] = \frac{Q^b_{ik} \cdot Q_{k+1,j}}{Q_{i,j}}$.

Now

$$\begin{aligned}
\sum_{G=(\dots)_k G'} d_{G'}(i,j)Pr[G|\xi[i\dots j]] &= \sum_{G'} \sum_{G''=(G''')_k} d_{G'}(i,j)Pr[G''G'|\xi[i\dots j]] \\
&= \sum_{G'} \sum_{G''=(G''')_k} d_{G'}(i,j)\frac{\exp(E(G'')/kT)\exp(G'/kT)}{Q_{ij}} \\
&= \sum_{G'} \sum_{G''=(G''')_k} d_{G'}(i,j)\frac{\exp(E(G'')/kT)\exp(G'/kT)}{Q_{ij}} \\
&= \sum_{G'} d_{G'}(i,j)\frac{\left(\sum_{G''=(G''')_k}\exp(E(G'')/kT)\right)\exp(G'/kT)}{Q_{ij}} \\
&= \sum_{G'} d_{G'}(i,j)\frac{Q^b_{i,k}\exp(G'/kT)}{Q_{ij}}
\end{aligned}$$

Now we can again simply extend by $Q_{k+1,j}$, getting

$$\begin{aligned}
&= \sum_{G'} d_{G'}(i,j)\frac{Q^b_{i,k}\cdot Q_{k+1,j}\cdot\exp(G'/kT)}{Q_{ij}\cdot Q_{k+1,j}} \\
&= \sum_{G'} d_{G'}(i,j)\frac{Q^b_{i,k}\cdot Q_{k+1,j}}{Q_{ij}}\cdot\frac{\exp(G'/kT)}{Q_{k+1,j}} \\
&= Pr[G=(\dots)_k G'|\xi[i\dots j]]\sum_{G'} d_{G'}(i,j)Pr[G'|\xi[k+1\dots j]] \\
&= Pr[G=(\dots)_k G'|\xi[i\dots j]]\cdot E[d_G(k+1,j)]
\end{aligned}$$

Overall we get

$$EL_{bp(k)} = (b+E[d_G(k+1,j)])\cdot Pr[G=(\dots)_k G'|\xi[i\dots j]]$$

and thus the second summand.

Appendix C: Notations

Table 1: **Basic notations**

Notations	Definitions
x	RNA sequence
$x[i..j]$	subsequence $x_i, x_{i+1}, \ldots, x_j$
G	secondary structure viewed as a outerplanar graph $G(V, E)$
V	vertex set of G
E	edge set of G
B	set of elements in E which are base pairs
B_k	set of base pairs enclosing k
$\{i, j\} \in B$	$\{i, j\}$ forms a base pair in G
$d_{v,w} = d$	distance between v and w in G is exactly d
$d^I_{v,w}$	inside distance between v and w in G
$d^O_{v,w}$	outside distance between v and w in G
n	length of the RNA sequence x
a	edge weight of a backbone edge in G
b	edge weight of a base pair edge in G
D	number of distances considered
c_b	$2b/lcd(a, b) + 1$

Table 2: **Notations of partition functions.** The (time) complexities are estimated under the assumption that positions of the start/end nucleotides v and w are given. †The complexity of $Z^{v,w}_{p,q}[d_O, d_I]$ is estimated under the assumption that the paritions $Z^{B,v}_{i,j}[d_\ell, d_r]$ for all i, j, d_ℓ and d_r have been pre-computated. ††The dominant complexity results from computating the partition function $Z^{B,v}_{i,j}[d_\ell, d_r]$.

Notation	Interval	Restrictions	Complexity	eqn.
Q	$[1, n]$	–	$O(n^4)$	[1]
$Z^I_{i,j}[d]$	$[i, j]$	$d_{i,j} = d$	$O(n^3 D)$	1
$Z^{I'}_{i,j}[d]$	$[i, j]$	$d_{i+1,j-1} = d$	$O(n^3 D)$	2
$Z^{v,w}_0[d]$	$[1, n]$	$d_{v,w} = d$ && $B_v \cap B_w = \emptyset$	$O(n^3 D^2)$	4
$Z^{v,w}_{p,q}[d_O, d_I]$	$[p, q]$	$\{p, q\} \in B$, $d^I_{v,w} = d_I$, $d^O_{v,w} = d_O$	$O(n^3 D^4)^{\dagger}$	8
$Z^{v,w}[d]$	$[1, n]$	$d_{v,w} = d$	$O(n^4 D^2 c_b^2)$	9
$Z^{B,v}_{i,j}[d_\ell, d_r]$	$[i, j]$	$\{i, j\} \in B$, $d_{v,i} = d_\ell$, $d_{v,j} = d_r$	$O(n^4 D^2 c_b^2)^{\dagger\dagger}$	10

References

[1] McCaskill, J.S.: The equilibrium partition function and base pair binding probabilities for RNA secondary structure. Biopolymers **29**(6-7), 1105–19 (1990)
